# Toward same-day detection of *Salmonella*: a rapid and cost-effective analytical method

**DOI:** 10.3389/fmicb.2025.1710697

**Published:** 2025-11-27

**Authors:** Daniela Cristiano, Maria Francesca Peruzy, Silvia Castellano, Andrea Mancusi, Yolande Thérèse Rose Proroga, Elisabetta Delibato, Roberta Mazzocca, Rosa Luisa Ambrosio, Nicoletta Murru

**Affiliations:** 1 Department of Food Microbiology, Istituto Zooprofilattico Sperimentale del Mezzogiorno, Naples, Italy; 2 Department of Veterinary Medicine and Animal Production, University of Naples Federico II, Naples, Italy; 3 Department of Food Safety, Nutrition and Veterinary Public Health, Istituto Superiore di Sanità, Rome, Italy; 4 Task Force on Microbiome Studies, University of Naples Federico II, Naples, Italy

**Keywords:** *Salmonella*, real-time PCR, minimum pre-enrichment time, leafy greens, minced meat, mozzarella cheese, mussel

## Abstract

**Introduction:**

Conventional methods for *Salmonella* detection in food samples are time-consuming, often requiring up to 5 days for pathogen identification. To prevent the distribution of contaminated foods on the market, rapid methods for early *Salmonella* detection are urgently needed, especially within the food industry, where timely responses are critical to public health and supply chain management. The aim of the present study was to evaluate a rapid strategy based on Real-Time PCR for detecting *Salmonella* in various food matrices.

**Methods:**

Several approaches were tested to rapidly detect *Salmonella* in experimentally contaminated leafy greens, minced meat, mozzarella cheese, and mussel samples. The protocols included two DNA extraction methods (boiling and Chelex 100), two enrichment broths (BPW and BPW supplemented with RAPID’*Salmonella* Capsules), and two incubation temperatures (37 °C and 42 °C).

**Results:**

Using preheated BPW at 41.5 °C overnight, *Salmonella* was detectable within 4 h when DNA was extracted via the Chelex 100 method.

**Discussion:**

The application of this rapid, automated, and low-cost analytical strategy could enable both food business operators and competent authorities to significantly enhance the control of food products. This method may represent an innovative tool for improving the assessment of epidemic outbreaks, ensuring not only food safety but also rapid diagnosis during emergencies.

## 1 Introduction

Each year, foodborne pathogens are responsible for over half of all food-related illnesses worldwide. Among these pathogens, *Salmonella* stands out as a major contributor, causing hundreds of thousands of deaths annually (Yang et al., 2018; Kim et al., 2021). In the European Union (EU), Salmonellosis is the second most frequently reported zoonosis and the major cause of foodborne outbreaks. In 2023, a total of 77,486 cases of human salmonellosis were reported in EU with a notification rate of 18.0 per 100,000 population (EFSA, 2024). In Italy, salmonellosis is the most reported foodborne infection, with over 3,500 cases reported annually (Leati et al., 2021). *Salmonella* infection in humans usually manifests as self-limiting gastroenteritis with nausea, abdominal cramps, vomiting, and diarrhea. Still, in some cases, it can be life-threatening, especially in young children (<5 years), the elderly, and immunocompromised adults (Liu et al., 2022).

The genus *Salmonella* consists of two species: *Salmonella enterica* and *Salmonella bongori*. *Salmonella enterica* (*S.*) can be further divided, into six subspecies and according to its surface antigens (O and H) into over 2,600 serotypes of which 99% are associated with animal and human infections (Ferrari et al., 2019). In 2023, the most commonly reported *Salmonella* serovars in human infections were *S. enteritidis*, *S. typhimurium* and monophasic *S. typhimurium*, 1,4,[5],12:i:-. *Salmonella* serovars live in the gastrointestinal tract of a wide range of domestic and wild animals. They can reach humans following direct contact with infected animals or *via* the consumption of contaminated foods. The pathogen can survive in all kinds of foods, e.g., meat, meat products, fish, fishery products, fruit, vegetables, spices, and herbs (Peruzy et al., 2022). The main sources of human infection are eggs and egg products, followed by pig meat and products thereof and bakery products (EFSA, 2022). However, a wide range of foods such as dairy products and vegetables has been implicated in salmonellosis. Several factors occurring from production to consumption, such as poor hygiene during slaughtering or harvesting, processing, transportation, and manipulation, may contribute to the survival of *Salmonella* in such foodstuffs (Ehuwa et al., 2021).

At the European level (EU), an integrated approach has been implemented for the control of *Salmonella* from farm to fork, with control programs conforming to European legislation (Wang et al., 2021). According to the Reg. (EC) 2073/2005 and any subsequent amendment, an absence of *Salmonella* is required in different food categories such as minced meat and meat preparation, dairy products, ready-to-eat vegetables and mussels. Pathogen isolation is generally performed through analytical reference methods which are reliable and sensitive but long and time-consuming. The ISO method (ISO 6579-1:2017/Amd 1:2020) for pathogen detection involves the use of different agar media after a pre-enrichment, in buffered peptone water and selective enrichment phases which allow the pathogen’s growth but prolong the detection time (Wang et al., 2021). Therefore, the whole process, considering also the biochemical and serological confirmation, takes up to at least 5 days to confirm the presence of the pathogen (Wang et al., 2021). The timing of analyses is incompatible with the need for early detection of *Salmonella* in food, to both intervene rapidly and prevent the distribution of contaminated food and in the event of epidemic outbreaks (Heymans et al., 2018). Molecular-based approaches have become increasingly important in microbial identification and diagnostics due to their high reliability, accuracy, and specificity (Awang et al., 2021). Among these, PCR-based methods have been extensively applied for the detection of *Salmonella* in pre-enriched food samples (Machado et al., 2019; Öz et al., 2020; Liu et al., 2022). However, these assays often remain time-consuming or technically demanding. For instance, Zhai et al. (2019) developed a real-time NASBA assay with high specificity and sensitivity for viable *Salmonella* cells, but requiring a 12-h pre-enrichment step. Conversely, Liu et al. (2022) proposed a CRISPR/Cas12a-based RPA assay suitable for on-site detection, yet its implementation involves complex optimization procedures and specialized reagents. Furthermore, many of these molecular assays have been optimized for a single food matrix, limiting their broader applicability. The present study aimed to develop a rapid and versatile qualitative Real-Time PCR method for *Salmonella* detection across different food matrices, with a total analysis time of approximately 7 h.

## 2 Materials and methods

### 2.1 Food samples for the detection of *Salmonella* in experimentally contaminated foods

Leafy greens (LG), minced meat (MM), water buffalo mozzarella cheese (WBMC), and mussels (M) samples were purchased in different supermarkets in the Campania Region (South Italy). All samples of around 200–300 g were transported at 4 °C to the laboratory within 1 h. Samples were analyzed before *Salmonella* strain’s inoculum with the ISO 6579-1:2017/Amd 1:2020 to evaluate the absence of the pathogen.

### 2.2 *Salmonella* strains

*Salmonella Typhimurium*, collected from the *Salmonella* Collection of the *Salmonella* Typing Center of the Campania Region (Department of Food Microbiology, Istituto Zooprofilattico Sperimentale del Mezzogiorno, Portici, NA, Italy), was used to contaminate food samples. Strains were cultured twice in nutrient agar (NA; CM0003, Oxoid, Basingstoke, UK) and incubated at 37 °C for 24 h. Colonies were then transferred in Buffered Peptone Water (BPW; CM0509, Oxoid, Basingstoke, UK). Contamination levels were determined by counting on Plate Count Agar (PCA; CM0325, Oxoid). Food samples were contaminated by dipping at low (1–10 CFU/25 g – LC) and high (10–10^2^ CFU/25 g – HC) levels.

### 2.3 Detection of *Salmonella* in experimentally contaminated samples by using different extraction methods

#### 2.3.1 Experimental contamination of the food matrix

Twenty-five grams of leafy green were placed in a sterile stomacher bag and homogenized for 3 min at 230 rpm using a peristaltic homogenizer (BagMixer®400 P, Interscience, Saint Nom, France) after the addition of 225 mL of sterile BPW. Each homogenate was inoculated with 1 mL of dilutions with the low and high levels as indicated in Section 2.2. Seeded samples were incubated at 37 °C for 20 h. During this phase, 2 mL of pre-enrichment media at different incubation times (0, 2, 4, 5, 6, 7, 8 and 20 h) were collected and subjected to DNA extraction.

#### 2.3.2 DNA extraction

DNA was extracted from each homogenate at time zero (T-0) and after 2 (T-2), 4 (T-4), 5 (T-5), 6 (T-6), 7 (T-7), 8 (T-8), and 20 (T-20) hours of incubation. DNA from leafy green homogenate was extracted by using the boiling (section 2.3.2.1) and Chelex 100 (section 2.3.2.1) methods.

##### 2.3.2.1 Boiling method

One mL of each homogenate was transferred into a 2 mL tube. The cell suspension was centrifuged for 10 min at 14,000 g. The supernatant was discarded carefully and the pellet was resuspended in 100 ul of DNase-RNase free distilled water by vortexing. The solution was incubated for 10 min at 100 °C and centrifuged for 5 min at 14,000 g at 4 °C. An aliquot of 5 μL of the supernatant was used as the template DNA in the real time PCR.

##### 2.3.2.2 Chelex 100 method

One or 10 mL of each homogenate were transferred into a 2 mL tube or 15 mL tube, and centrifuged for 10 min at 10,000 × g at 4 °C. The supernatant was discarded, and the pellet was re-suspended in 300 μL of 6% Chelex 100 by vortexing and incubated at 56 °C for 20 min and further 8 min at 100 °C. The suspension was immediately chilled on ice for 1 min, and centrifuged for 5 min at 10,000 g at 4 °C. An aliquot of 5 μL of the supernatant was used as the template DNA in the real time PCR.

#### 2.3.3 Real-time PCR (qPCR)

To evaluate the presence of *Salmonella*, the *iQ-Check Real-Time PCR Kit* (Bio-Rad, Hercules, CA, United States) was used following the manufacturer’s recommendations. The assay is based on the amplification and detection of a specific *Salmonella* spp. DNA target using fluorescent probes. Each reaction was prepared by combining 5 μL of extracted DNA with 45 μL of PCR master mix, for a total reaction volume of 50 μL. Amplifications were carried out with a total run time of approximately 1 h 10 min.

Fluorescence data were analyzed using the Bio-Rad CFX Manager software, and results were interpreted following the manufacturer’s criteria, taking into account the amplification curve profile and the internal control response. Samples were considered positive when the specific amplification curve was observed with a Ct value ≤ 45 and a valid internal control signal. Late Ct values approaching the assay cut-off were interpreted as indicative of a very low concentration of target DNA. This interpretation is consistent with the analytical sensitivity and detection limits established in the AFNOR-certified and ISO 16140-2 validation study of the method.

### 2.4 Detection of *Salmonella* in experimentally contaminated samples by using different broths and incubation temperatures

#### 2.4.1 Experimental contamination of the food matrix

Twenty-five grams of leafy green, minced meat, and water buffalo mozzarella cheese samples were placed in sterile stomacher bags and homogenized for 3 min at 230 rpm using a peristaltic homogenizer (BagMixer®400 P, Interscience, Saint Nom, France) after the addition of 225 mL of BPW. For half of the samples, a RAPID’S *Salmonella* Capsule (3564710, Bio-Rad, Hercules, California, United States) was added to the BPW (BPWC). Each homogenate was inoculated with 1 mL of low- and high-level dilutions as described in Section 2.1. Homogenates containing the RAPID’S *Salmonella* Capsule were incubated at 42 °C, whereas those without the capsule were incubated at 37 °C, both for 20 h. During incubation, aliquots of pre-enrichment media were collected at various time points and subjected to DNA extraction (see Section 2.4.2).

#### 2.4.2 DNA extraction and real-time PCR

DNA was extracted from 20 mL of each homogenate at time zero (T-0) and after 2 (T-2), 4 (T-4), 5 (T-5), 6 (T-6), 7 (T-7), 8 (T-8), and 20 (T-20) hours of incubation by using the Chelex 100 (section 2.3.2.2) method. Real-time PCR was performed by using the “iQ-Check Real-Time PCR Kit” (section 2.3.3).

### 2.5 Detection of *Salmonella* in experimentally contaminated samples by using pre-heated BPW

#### 2.5.1 Experimental contamination of the food matrix

Twenty-five grams of leafy green, minced meat, water buffalo mozzarella cheese, and mussels samples were placed in a sterile stomacher bag and homogenized for 3 min at 230 rpm using a peristaltic homogenizer (BagMixer®400 P, Interscience, Saint Nom, France) after the addition of 225 mL of sterile overnight-preheated Buffered Peptone Water (BPW; CM0509, Oxoid, Basingstoke, UK). Before use, BPW was heated overnight at 41.5 °C.

Each homogenate was inoculated with 1 mL of dilutions with the low and high levels as indicated in Section 2.1. Seeded samples were incubated at 37 °C for 20 h.

#### 2.5.2 DNA extraction and real-time PCR

DNA was extracted from 1 of each homogenate after 4 (T-4), 5 (T-5), 6 (T-6), and 20 (T-20) hours of incubation by using the Chelex 100 (section 2.3.2.2) method. Real-time PCR was performed by using the “iQ-Check Real-Time PCR Kit” (section 2.3.3).

### 2.6 Statistical analysis

All quantitative PCR (qPCR) data were statistically analyzed to assess the influence of the inoculum level, extraction method, and incubation time on *Salmonella* detection efficiency. Cycle threshold (Ct) values were considered continuous variables indicative of the amount of amplifiable target DNA.

Prior to inferential testing, the data were examined for normality (Shapiro–Wilk test) and homogeneity of variance (Levene’s test). When these assumptions were met, two-way analysis of variance (ANOVA) was used to evaluate the main effects of inoculum level (low vs. high) and extraction method (Boiling vs. Chelex-100), including their interaction. For non-normally distributed data, equivalent non-parametric Kruskal–Wallis tests were applied.

Pairwise comparisons between extraction methods within each inoculum level were performed using paired Student’s *t*-tests, based on Ct values measured at identical incubation times. In a separate analysis, the effect of sample volume (1 mL vs. 10 mL of homogenate) extracted using the Chelex-100 method was similarly evaluated by paired *t*-tests to determine whether increasing the extraction volume improved detection sensitivity.

The relationship between incubation time and Ct decrease was modeled using simple linear regression (*Ct* = β₀ + β₁·time), and regression slopes were compared between treatments to estimate the rate of DNA detection improvement over time. Descriptive statistics (mean ± SD) were computed for each condition, and data visualization was performed through ggplot2 in R software (v.4.3.0; R Foundation for Statistical Computing, Vienna, Austria). Statistical significance was set at *p* < 0.05.

## 3 Results

### 3.1 Early detection of *Salmonella* in spiked leafy greens using alternative DNA extraction methods

In the first trial, both the boiling and chelex-100 protocols were employed to extract bacterial DNA from 1 mL of homogenized leafy green samples. Both methods proved effective for the early detection of *Salmonella* in vegetables, with positive results obtained from the 5th hour of incubation at the lowest contamination level (0–10 CFU/25 g) and from the 4th hour at the highest level (10–10^2^ CFU/25 g), respectively (Table 1).

**Table 1:** Detection of *Salmonella* by using the Boiling and Chelex 100 method from 1 mL of homogenate of leafy greens artificially contaminated with two bacterial levels (LC, low = 1–10 CFU/25 g; HC, high = 10–10^2^ CFU/25 g) and incubated for 20 h.

Inoculum level	Incubation temperature	Hours of incubation	qPCR		ΔCt (Chelex–Boiling)	Significance
			Boiling (C_t_)	Chelex (C_t_)		
LC	37 °C	0	–	–		
		2	–	–		
		4	–	–		
		5	39.66	38.73	−0.93	n.s.
		6	36.55	37.87	+1.32	n.s.
		7	34.1	35.57	+1.47	n.s.
		8	31.86	34.11	+2.25	n.s.
		20	21.08	21.99	+0.91	n.s.
HC		0	–	–		
		2	–	–		
		4	39.64	39.81	+0.17	n.s.
		5	34.14	38.26	+4.12	*P* < 0.05
		6	33.34	35.75	+2.41	*P* < 0.05
		7	29.69	34.16	+4.47	*P* < 0.05
		8	27.76	31.42	+3.66	*P* < 0.05
		20	17.8	18.4	+0.60	n.s.

C_t_, Cycle threshold; n.s., not significant.

Cycle threshold (Ct) values decreased progressively with incubation time across all samples, indicating a time-dependent increase in *Salmonella* DNA detectability. Samples inoculated at high contamination levels (HC) exhibited consistently lower Ct values compared to low contamination (LC) samples, confirming faster detection at higher bacterial loads.

Between extraction methods, the boiling protocol yielded slightly lower mean Ct values than the chelex-100 method in both LC and HC samples (mean ΔCt = +0.6 and +1.9, respectively). This difference was statistically significant only at the high contamination level (*t* = 3.32, *p* = 0.02). Linear regression analysis showed a comparable overall decrease of approximately 2 Ct units per hour for all treatments, with Boiling displaying marginally steeper slopes, suggesting a faster DNA detection rate.

Leafy greens were again used as the food matrix in a subsequent trial, where DNA extraction was performed from 10 mL of the incubated homogenate instead of 1 mL. However, the detection time remained unchanged, with *Salmonella* being identified at 4 h for the highest inoculation level and 5 h for the lowest (Table 2). Comparison of extraction volumes revealed that using 10 mL of homogenate yielded slightly lower mean Ct values than 1 mL (ΔCt ≈ 0.4), but the difference was not statistically significant for either contamination level (LC: *t* = 1.06, *p* = 0.35; HC: *t* = 0.96, *p* = 0.38). Linear regression analysis showed parallel trends for both extraction volumes (slope ≈ − 2 Ct h^−1^), indicating that increasing the extracted volume did not appreciably enhance the detection rate of *Salmonella* under the tested conditions.

**Table 2:** Detection of *Salmonella* by using the Chelex 100 method from 10 mL of homogenate of leafy greens artificially contaminated with two bacterial levels (LC, low = 1–10 CFU/25 g; HC, high = 10–10^2^ CFU/25 g) and incubated for 20 h.

Leafy green samples			
Inoculum level	Incubation temperature	Hours of incubation	qPCR (C_t_)
LC	37 °C	0	–
		2	–
		4	–
		5	38.87
		6	38.96
		7	35.02
		8	32.53
		20	20.92
HC		0	–
		2	–
		4	40.09
		5	39.12
		6	34.8
		7	32.5
		8	28.69
		20	18.19

C_t_, Cycle threshold.

### 3.2 Influence of broth composition and incubation temperature on the detection of *Salmonella* in spiked food samples

An additional strategy employed in the present study involved modifying the enrichment broth formulations and varying the incubation temperature. RAPID’S *Salmonella* capsules, which are selective supplements, were added to half of the samples prepared in BPW. In this trial, DNA extraction was performed using 20 mL of each incubated homogenate and the Chelex-100 method. The results obtained from homogenates incubated with the capsule are not shown, as the detection of positive samples was inconsistent and exhibited no clear temporal trend.

#### 3.2.1 Detection in minced meat

In minced meat, *Salmonella* was detected from the 8th hour of incubation onwards in samples seeded at the lowest contamination level. In samples with the highest contamination level, detection occurred from the 6th hour onwards (Table 3).

**Table 3:** Detection of *Salmonella* by using Chelex100 extraction method from 20 mL of homogenate of minced meat, leafy greens and water buffalo mozzarella cheese artificially contaminated with two bacterial levels (LC, low = 1–10 CFU/25 g; HC, high = 10–10^2^ CFU/25 g) and incubated in Buffered Peptone Water (BPW) 20 h at 37 °C.

Food samples	Enrichment medium	Inoculum level	Hours of incubation	qPCR (C_t_)
Minced meat	BPW	LC	0	–
			2	–
			4	–
			5	–
			6	–
			7	–
			8	37.6
			20	29.66
		HC	0	–
			2	–
			4	–
			5	–
			6	40.91
			7	39.51
			8	38.16
			20	33.91
Leafy green	BPW	LC	0	–
			2	–
			4	–
			5	40.91
			6	39.76
			7	36.21
			8	33.88
			20	26.62
		HC	0	–
			2	–
			4	37.96
			5	31.48
			6	38.04
			7	32.78
			8	31.59
			20	25.86
Water buffalo mozzarella cheese	BPW	LC	0	–
			2	–
			4	–
			5	–
			6	40.04
			7	39.67
			8	35.21
			20	20.56
		HC	0	–
			2	–
			4	36.02
			5	39.61
			6	35.75
			7	34.21
			8	30.23
			20	16.39

C_t_, Cycle threshold.

#### 3.2.2 Detection in leafy greens

In leafy green samples, *Salmonella* was detected from the 5th hour of incubation onwards in samples seeded at the lowest contamination level, whereas in samples with the highest contamination level, detection occurred from the 4th hour onwards (Table 3).

#### 3.2.3 Detection in water buffalo mozzarella cheese

In water buffalo mozzarella cheese, *Salmonella* was detected from the 6th hour of incubation onwards in samples seeded at the lowest contamination level, whereas in samples with the highest contamination level, detection occurred from the 4th hour onwards (Table 3).

### 3.3 Enhanced detection of *Salmonella* in spiked food samples using pre-heated buffered peptone water

In the final trial, BPW was preheated to 41.5 °C prior to sample inoculation. *Salmonella* was detected from the 4th hour of incubation in minced meat, mozzarella, and mussel samples, regardless of the bacterial load. In leafy green samples, detection failed only in the case of low-level contamination (Table 4).

**Table 4:** Detection of *Salmonella* from 1 mL of minced meat (MM), leafy greens (LG), water buffalo mozzarella cheese (WBMC) and mussels (M) homogenates artificially contaminated with two bacterial levels (LC, low = 1–10 CFU/25 g; HC, high = 10–10^2^ CFU/25 g CFU/g) and incubated in preheated buffered peptone water (41.5 °C) by using Chelex100 extraction method.

Medium temperature	Inoculum level	Incubation temperature	Hours of incubation	qPCR (C_t_)			
				1 ml			
				MM	LG	WBMC	M
41.5 °C	LC	37 °C	4	39.51	–	35.06	43.02
			5	38.19	41.73	38.74	40.9
			6	39.74	38.31	38.55	39.12
			20	39.37	27.28	24.57	28.77
	HC		4	38.64	41.37	37.69	42.56
			5	38.9	37.27	36.54	36.36
			6	39.59	35.22	39.12	38.26
			20	25.33	25.16	22.45	32.12

C_t_, Cycle threshold.

## 4 Discussion

Over the past few years, various cultural and molecular methods have been employed for the detection of *Salmonella* in food. Although these approaches are reliable, they are often lengthy and time-consuming (Machado et al., 2019; Liu et al., 2022). Conventional microbiological techniques typically require up to 4 days to confirm negative results and 6–7 days to identify positive isolates (Zhai et al., 2019). Compared with traditional plating methods, PCR offers greater speed and specificity, enabling the detection of sub-dominant bacterial populations directly in food samples or after enrichment (Cremonesi et al., 2014). However, the inclusion of an enrichment step has been shown to improve detection sensitivity by diluting inhibitory substances and enhancing *Salmonella* growth, which is why enrichment-based approaches remain the most widely used in routine testing (Dmitric et al., 2018). In the present study, several trials were conducted to evaluate these aspects.

### 4.1 Effectiveness of different DNA extraction protocols for the identification of *Salmonella* in artificially contaminated matrices

PCR-based methods require efficient DNA extraction protocols for accurate pathogen detection. Efficient extraction is particularly crucial in complex matrices, as inhibitory substances may compromise amplification (Kasturi and Drgon, 2017). In this study, two non-solvent, cost-effective, and rapid DNA extraction methods—boiling and Chelex-100—were evaluated. Commercial DNA extraction kits were not considered due to their high cost and operational complexity. Instead, the focus was placed on the boiling and Chelex-100 methods as practical alternatives for routine use in diagnostic applications (Pacheco et al., 2023).

In the first trial, both methods were effective for the early detection of *Salmonella* in vegetables, starting from the 5th hour of incubation at the lowest contamination level (1–10 CFU/25 g) and from the 4th hour at the highest level (10–10^2^ CFU/25 g). This demonstrates that both extraction protocols are sufficiently sensitive to allow early pathogen recovery even in minimally contaminated matrices.

In an attempt to reduce the detection time, DNA extraction was performed using 10 mL of incubated homogenate instead of 1 mL. However, the detection time remained unchanged, with *Salmonella* being detected at 4 h for the highest inoculation level and 5 h for the lowest. This suggests that, at least for this matrix, increasing the extraction volume does not significantly enhance the sensitivity or accelerate detection. The comparison between DNA extraction volumes revealed that increasing the aliquot of homogenate from 1 mL to 10 mL did not significantly improve *Salmonella* detection efficiency when using the Chelex-100 extraction protocol. Although Ct values obtained from 10 mL samples were, on average, slightly lower than those from 1 mL samples (ΔCt ≈ 0.4), this difference was not statistically significant for either contamination level. This suggests that the extraction efficiency of the Chelex-100 method is not strongly dependent on the sample volume, at least within the tested range.

The absence of a clear improvement may be attributed to the limited recovery capacity of the resin and the saturation of chelating sites when processing larger sample volumes. Additionally, since both low and high contamination levels exhibited comparable time-to-detection trends, the total amount of target DNA present in 1 mL of enriched homogenate was apparently sufficient for reliable amplification.

These results support the use of smaller extraction volumes for routine qPCR detection, offering practical advantages in terms of reagent savings, reduced processing time, and simpler workflow without compromising analytical sensitivity. However, further testing on different food matrices or at lower contamination levels could clarify whether larger extraction volumes may confer benefits under more challenging detection conditions.

Although both the boiling and Chelex-100 methods yielded comparable results in this study, previous reports—such as that by Mohammadi et al. (2017)—have demonstrated that the Chelex-100 method produces higher-quality DNA. This resin exhibits a high affinity for polyvalent metal ions. During the extraction process, Chelex-100 promotes cell lysis through heat treatment, releasing DNA while simultaneously preventing its degradation by chelating metal ions that could otherwise catalyze nucleic acid hydrolysis (Yang et al., 2024). Although DNA quality was not directly assessed in our experiments, the superior performance reported in the literature led us to select the Chelex-100 method for subsequent analyses to ensure greater reliability and consistency.

### 4.2 Impact of broth formulations and incubation temperatures on *Salmonella* detection in experimentally contaminated samples

An alternative approach to reducing detection time involved modifying the enrichment broth formulations and incubation temperatures. Specifically, RAPID’S *Salmonella* capsules, used as selective supplements, were added to half of the samples prepared in BPW. Following the manufacturer’s recommendations for *Salmonella* recovery, homogenates containing the capsule were incubated at 42 °C. The results from these samples are not presented, as the detection of positive cases was sporadic and showed no consistent temporal pattern.

Overall, modifying incubation temperature and incorporating selective supplements influenced *Salmonella* detection. Although *S. typhimurium* has been reported to be thermotolerant at 45 °C (Pesingi et al., 2017), and elevated temperatures have been associated with a reduction in background flora (Daquigan et al., 2016), incubation at 42 °C did not improve detection efficiency in the present trials (Data not shown). Conversely, the standard incubation temperature of 37 °C, as recommended by ISO 6579-1:2017/Amd 1:2020, resulted in faster and more reliable pathogen recovery across these matrices. The diverse and complexity of food matrices pose great challenges for rapid and ultra-sensitive detection of Salmonella in food samples (Vinayaka et al., 2019).

In minced meat samples enriched in BPW, *Salmonella* was detected as early as 8 h post-incubation at the lowest contamination level, consistent with Josefsen et al. (2007) and Krämer et al. (2011), who reported a minimum incubation time of approximately 8 h for reliable PCR detection in meat products. At the highest contamination level, detection occurred earlier, at 6 h. In contrast, earlier detection was achieved in both leafy greens and water buffalo mozzarella cheese across contamination levels. For leafy green samples, increasing the homogenate volume used for DNA extraction to 20 mL enabled detection of *Salmonella* at the lowest contamination level from 6 h of incubation; however, recovery was not improved compared to smaller volumes (1 mL or 10 mL) and was slightly delayed (6 h versus 5 h). These findings suggest that matrix effects and bacterial distribution exert a greater influence on detection efficiency than absolute bacterial load in these food types.

### 4.3 Rapid detection of *Salmonella* in inoculated food samples using pre-heated BPW

In the present study, BPW was preheated to 41.5 °C. The use of preheated broth had a notable impact on *Salmonella* growth, enabling its detection after only a few hours of incubation. *Salmonella* was consistently detected in minced meat, mozzarella, and mussels starting from the 4th hour, regardless of bacterial load used (1 mL). In leafy greens, detection failed only when 1 mL of homogenate was used for low-level contamination at the 4-h mark.

The entire *Salmonella* detection process in the present study was completed in under 7 h, involving minimal handling and requiring no more than 4 h of culture enrichment. Notably, *Salmonella* was detectable even at low concentrations, which is critical given that ingestion of a single colony-forming unit (CFU) can potentially lead to infection (Wang et al., 2021).

Many recently proposed methods include short enrichment phases (depending on the food matrix) but involve complex sample preparation procedures that ultimately prolong the overall detection time (Wang et al., 2018; Du et al., 2021; Liu et al., 2022). In contrast, the protocol developed in the present study ensures a streamlined and time-efficient approach, combining simplified sample handling with rapid diagnostic output. qPCR was used as a qualitative method for *Salmonella* detection, offering enhanced sensitivity, speed, and safety compared to traditional endpoint PCR, which typically requires post-amplification steps, thereby providing a more practical and reliable tool for routine analysis (Machado et al., 2019; Sahu et al., 2019; Vinayaka et al., 2019; Peruzy et al., 2020). By integrating minimal enrichment time with qPCR and straightforward processing, the present study provides a practical and robust solution for the rapid detection of *Salmonella* in diverse food matrices.

## 5 Conclusion

In conclusion, this study combined multiple pre-detection strategies—including the use of different pre-enrichment broths, DNA extraction methods and enrichment volumes—with a molecular detection kit to establish a rapid and robust analytical workflow.

By preheating BPW at 41.5 °C overnight and extracting DNA using the Chelex 100 method, *Salmonella* was detectable as early as 4 h post-incubation. This approach enables pathogen detection in less than 7 h.

To our knowledge, this is the first study demonstrating the effectiveness of a single rapid protocol across a broad range of experimentally contaminated food matrices, including meat, cheese, vegetables, and mussels.

The key advantages of this method are its automation potential and suitability for high-throughput testing. The results presented here may support the implementation of a rapid, automated, and cost-effective strategy for routine *Salmonella* monitoring across various food supply chains. Furthermore, this approach could serve as a valuable tool for timely outbreak investigations, enhancing both food safety and emergency diagnostic response.

## Data Availability

The raw data supporting the conclusions of this article will be made available by the authors, without undue reservation.
